# Hallux valgus surgery in the public health system of Brazil

**DOI:** 10.31744/einstein_journal/2025AO1053

**Published:** 2025-09-11

**Authors:** Celso Garreta Prats Dias, Nelson Wolosker, Caio Augusto de Souza Nery, Marcelo Fiorelli Alexandrino da Silva, Mario Lenza

**Affiliations:** 1 Hospital Israelita Albert Einstein São Paulo SP Brazil Hospital Israelita Albert Einstein, São Paulo, SP, Brazil.

**Keywords:** Foot, Osteotomy, Public health, Mortality, Hallux valgus, Orthopedic procedures, Cost of illness

## Abstract

**Objective:**

This study aimed to describe patient profiles and analyze costs, hospital stay, and mortality over the past 17 years.

**Methods:**

This ecological study was conducted using data available on TabNet, a DATASUS platform. Public data (from the government health system) on hallux valgus surgeries performed in Brazil between 2008 and 2024 were extracted. Sex, age, municipal region, costs, length of stay, and death rates were analyzed.

**Results:**

We analyzed 37,741 hallux valgus surgeries, of which nine (0.024%) resulted in death. Most participants were female aged 40–69 years, with a mean length of stay of 1.7 days. The total cost averaged US$ 141.53 per participant.

**Conclusion:**

Hallux valgus surgery is a safe procedure with a low total cost, a short length of stay, and a low mortality rate.

**Level of evidence 4, Case Series.**

## Highlights

■Public hospitals in Brazil performed 37,741 hallux valgus surgery procedures between January 2008 and October 2024.

■The study population consisted of 79.7% females, with the most common age group being 40-69 years (67.7%). The mean length of stay was 1.7 days.

■The study population consisted of 79.7% females, with the most common age group being 40-69 years (67.7%). The mean length of stay was 1.7 days.

■The mean cost per patient was US$ 141.53.

■The overall mortality count was nine, corresponding to a death rate of 0.024%.

## In Brief

This retrospective study design can retrieve thorough information regarding specific conditions. No other kind of study is capable of evaluating thousands of procedures simultaneously with little to no harm inflicted on individual participants, as the data are accessed without liability to personal information. TABNET, a DataSUS platform, is a reliable source of information for investigating demographic data and treatment outcomes.

## Graphical abstract

**Figure:**
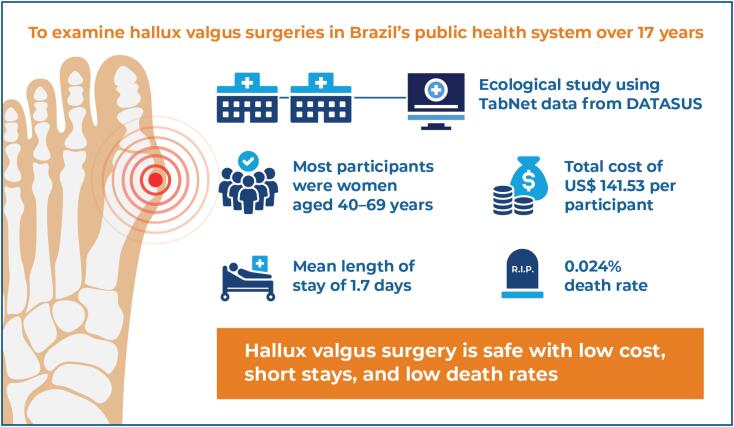

## INTRODUCTION

Hallux valgus is a prevalent foot deformity that occurs in approximately 23% of younger adults and 35.7% of adults over 65 years old.^(1)^ Hallux valgus surgery (HVS) is one of the most common elective procedures on the forefoot.^(1,2)^ It is typically performed through a 5 cm incision and involves an osteotomy (cutting of the metatarsal bone) to correct the deformity.^(1,3)^ In HVS, it has become progressively more common to include a metal screw to help stabilize the osteotomy.^(3)^ Furthermore, new studies are evaluating the possibility of achieving similar or improved results through smaller incisions (known as minimally invasive or percutaneous procedures).^(3,4)^ Other surgical procedures that may be performed alone or in combination with metatarsal osteotomy include soft-tissue procedures, osteotomy of the proximal phalanx, fusion, or resection arthroplasty.^(1,5,6)^

Hallux valgus surgery aims to alleviate pain, improve function, enhance the quality of life, and mitigate the risk of falling, which carries a high risk of long bone fractures, hospitalization, and death.^(1,5)^ Nevertheless, surgery can have associated risks, including a prolonged hospital stay,^(5)^ adverse events,^(6)^ and increased mortality.^(7)^ Furthermore, some studies have suggested that HVS incurs high hospitalization costs.^(8)^

Considering the clinical and economic relevance of HVS, it is essential to identify and evaluate the prevalence of complications and costs to the public health system in Brazil.^(9)^

## OBJECTIVE

This study aimed to describe patient profiles and analyze costs, hospital stay, and mortality over the past 17 years.

## METHODS

All patient data were obtained from the DATASUS Hospital Information System (SIH - *Sistema de Informações Hospitalares*), a Brazilian Ministry of Health data platform that gathers information on hospitalizations funded by the country's Brazilian Public Health System (SUS - *Sistema Único de Saúde*).

The study was approved by the Research Projects Management System (SGPP - *Sistema Gerenciador de Projetos de Pesquisa*; protocol: 5887), and a waiver of consent was granted.

### Procedure analysis

The following codes were used: Hallux valgus treatment involving first metatarsal osteotomy – 04.08.05.065-9; Hallux valgus treatment without first metatarsal osteotomy – 04.08.05.091-8 and, Osteotomy of the hand or foot – 04.08.06.018-2.

## RESULTS

In total, 37,741 HVS procedures were performed in Brazil between January 2008 and October 2024 (Table 1). We observed a 7.37% increase in the number of HVS procedures between 2008 and 2019, the year before the pandemic restrictions began to decrease the annual number of procedures. Regarding the country's regions, we observed that a higher proportion of patients were subjected to HVS in the southeastern region (49.4%), followed by the southern (34.2%), northeastern (7.3%), central-western (6.6%), and northern (2.5%) regions of Brazil. Females accounted for 79.7% of the study population, with the most common age group being 40–69 years (67.7%). The mean length of stay was 1.7 days.

**Table 1:** Patients subjected to hallux valgus surgeries in Brazil's public hospitals

Period	Total	Central-West	North-East	North	South-East	South
2008	2,403	122	270	131	1,153	727
2009	2,360	127	227	82	1,121	803
2010	2,254	139	231	76	1,060	748
2011	2,340	145	241	68	1,142	744
2012	2,256	138	210	66	994	848
2013	2,391	265	209	69	1,036	812
2014	2,394	215	161	73	1,075	870
2015	2,311	145	109	55	1,238	764
2016	2,270	173	117	38	1,249	693
2017	2,412	139	109	30	1,253	881
2018	2,500	102	153	22	1,306	917
2019	2,580	144	174	42	1,327	893
2020	1,297	123	102	27	620	425
2021	1,090	97	74	28	563	328
2022	2,055	145	98	41	1,037	734
2023	2,349	139	135	41	1,209	825
2024	2,479	124	148	38	1,277	892
Total	3,7741	2,482	2,768	927	18,660	12,904
Deaths (n)	9	0	0	4	3	2
Costs (US$)	5,341,8 44.60	336,514 .82	333,199.73	104,728.58	2,670,233.52	1,897,167.94
Cost per patient (US$)	141.53	135.85	120.37	112.98	143.10	147.02

The mean cost per patient was US$ 141.53 (Table 1). The total cost of HVS was higher in the southwestern region (US$ 2,670,233.52), where there was also the greatest number of procedures (n=18,660). This is the most populous region in the country, with 84.8 million people, equivalent to 41.8% of Brazil's total population.

The overall mortality count was nine, corresponding to a death rate of 0.024%. Mortality per region was 0.015% in the southern region (14.74% of Brazil's total population), 0.016% in the southeastern region (41.78% of Brazil's total population), and 0.431% in the northern region (8.54% of Brazil's total population); no cases were observed in the central-western and northeastern regions (8.02% and 26.91% of Brazil's total population, respectively). The highest absolute mortality per region following HVS was reported in the north (four patients). However, the northern region is the second least populated region and had the fewest procedures (n=927). The relative risk of death in the northern region was 18 times higher than that in Brazil (p<0.005). The northern region was the least expensive region for performing HVS with public resources in Brazil (p<0.005), with an average cost of US$ 112.98 per patient.

The death count per year was higher in 2008, the first year for which data were available for this study. There were two deaths between May and June in the southern region and one in August in the northern region. One-third of the deaths occurred in 2008, with statistical significance (p<0.05) compared to the total mortality.

## DISCUSSION

We evaluated information on primary and elective HVS in public hospitals in Brazil between January 2008 and October 2024 using data from 37,741 procedures.

The preoperative demographic data reported in this study are compatible with the current literature.^(1-3)^ Most participants were female, ranging from 40 to 69 years of age, with a length of stay of <2 days.

The total number of HVS procedures was one per 100,000 per year, which is lower than the rate reported abroad.^(10)^ The mortality rate related to HVS in this study was 0.024%, which is similar to that in Jameson's review,^(7)^ reporting a mortality rate of 0.04% related to first metatarsal osteotomies. The southern and southeastern regions had a combined mortality rate of 0.016%. However, in the central-western and northeastern regions, the mortality rate could not be calculated because no deaths were reported. Mortality in the northern region was higher than expected, which may be attributed to the limitations of this retrospective study. Because the data were anonymized, we did not have access to patients’ medical records, restricting our ability to assess potential confounding factors. The cause of death was not necessarily related to the surgery itself but potentially to other associated conditions present at the time of surgery, not specified by the International Classification of Diseases code assigned for hospitalization.

The most populated regions of Brazil have more procedures per population and higher monetary investment for treating hallux valgus surgically, as expected since HVS is an elective procedure. The least populated regions had a lower cost of public resources for treating hallux valgus; however, the region with the lowest number of HVS procedures and the least investment for treating this disease had the highest mortality rate. The reasons for this difference are unknown; it could be inferred that a higher investment per procedure may have been a protective factor, which a myriad of reasons, such as enhanced prophylaxis for infection and sepsis or better intensive care unit installations, could explain.

A considerable decrease was observed in the average number of procedures per year since the first year of the COVID-19 pandemic, from 2,373 HVS in 2019 to 1854 since 2020, representing a decrease of 21.85%. However, there were no deaths after 2016; therefore, the impact on the mortality rate of HVS related to COVID-19 contamination surges is unknown.

This retrospective study has limitations related to its design, including reporting bias from large databases and limited control over the variables. However, no other type of study is capable of evaluating thousands of procedures simultaneously, with minimal harm inflicted on individual participants, since the data are accessed without liability to personal information. Future studies should focus on mortality rates and other important information.

## CONCLUSION

Hallux valgus surgery is a safe procedure with a low total cost, a short length of stay, and a low mortality rate.
